# Monitoring attentional state with fNIRS

**DOI:** 10.3389/fnhum.2013.00861

**Published:** 2013-12-13

**Authors:** Angela R. Harrivel, Daniel H. Weissman, Douglas C. Noll, Scott J. Peltier

**Affiliations:** ^1^Bioscience and Technology Branch, NASA Glenn Research CenterCleveland, OH, USA; ^2^fMRI Laboratory, Department of Biomedical Engineering, University of MichiganAnn Arbor, MI, USA; ^3^Department of Psychology, University of MichiganAnn Arbor, MI, USA

**Keywords:** near infra-red spectroscopy, attention, default mode network, classification, human performance

## Abstract

The ability to distinguish between high and low levels of task engagement in the real world is important for detecting and preventing performance decrements during safety-critical operational tasks. We therefore investigated whether functional Near Infrared Spectroscopy (fNIRS), a portable brain neuroimaging technique, can be used to distinguish between high and low levels of task engagement during the performance of a selective attention task. A group of participants performed the multi-source interference task (MSIT) while we recorded brain activity with fNIRS from two brain regions. One was a key region of the “task-positive” network, which is associated with relatively high levels of task engagement. The second was a key region of the “task-negative” network, which is associated with relatively low levels of task engagement (e.g., resting and not performing a task). Using activity in these regions as inputs to a multivariate pattern classifier, we were able to predict above chance levels whether participants were engaged in performing the MSIT or resting. We were also able to replicate prior findings from functional magnetic resonance imaging (fMRI) indicating that activity in task-positive and task-negative regions is negatively correlated during task performance. Finally, data from a companion fMRI study verified our assumptions about the sources of brain activity in the fNIRS experiment and established an upper bound on classification accuracy in our task. Together, our findings suggest that fNIRS could prove quite useful for monitoring cognitive state in real-world settings.

## Introduction

The ability to distinguish between high and low levels of task engagement is important for detecting and preventing performance decrements during safety-critical operational tasks in the real world. Examples of such tasks include commercial aviation, monitoring for air traffic control, executing space walks, performing surgery, and driving. Since accident-causing errors can be made even by skilled professionals (Dismukes et al., 2007), the ability to monitor cognitive state measures for low levels of task engagement in real time could be useful for developing an “early warning system” for detecting and preventing performance errors before they occur.

The use of cognitive state measures to optimize human performance (for example by informing flight automation or the operator themselves of a hazardous state) has been of particular importance to aviation safety (Pope et al., 1995; Schnell et al., 2004) and to the Augmented Cognition program (Raley et al., 2004) of the Defense Advanced Research Projects Agency of the United States. Additionally, such research is highly relevant to space flight, since adaptation to microgravity can cause performance decrements due to motion sickness, lack of sleep, loss of sensorimotor control, increased stress or mood changes (Cowings et al., 2003). More generally, the ability to monitor cognitive state for low levels of task engagement could be helpful for detecting and preventing vigilance decrements due to sleep-deprivation (Drummond et al., 2005; De Havas et al., 2012) or distraction (Strayer et al., 2011).

Monitoring brain activity may provide an effective means for monitoring cognitive state, and in particular for distinguishing between high and low levels of task engagement. Numerous functional magnetic resonance imaging (fMRI) studies have revealed that activity increases in a so-called “task-positive” network, which includes the dorsolateral prefrontal cortex (DLPFC), dorsal anterior cingulate cortex (dACC), superior and inferior parietal lobe (SPL and IPL), and anterior insula (AI), when participants perform a task as compared to when they rest (MacDonald et al., 2000; McKiernan et al., 2003; Dosenbach et al., 2006). In contrast, activity increases in a so-called “task-negative” network, which includes the anterior medial frontal gyrus (aMFG), posterior cingulate cortex (PCC), and certain regions of lateral parietal cortex (LPC), when participants rest as compared to perform a task (Raichle et al., 2001; Greicius et al., 2003). In other words, activity in the “task-positive” and “task-negative” networks is negatively correlated (Fox et al., 2005; Kelly et al., 2008). For this reason, monitoring activity in key regions of the “task-positive” network alone or monitoring activity in key regions of the “task-positive” and “task-negative” networks together may distinguish between relatively high and relatively low levels of task engagement (Drummond et al., 2005; Weissman et al., 2006; Chee et al., 2008). Given recent data indicating that interactions between key regions of the “task-positive” and “task-negative” networks vary with task engagement (Prado and Weissman, 2011), we predicted that either approach for monitoring brain activity would allow us to distinguish between relatively high and relatively low levels of task engagement, but that the latter approach would likely prove most effective.

Since hemodynamic activity cannot be monitored with fMRI outside of a laboratory, we employed functional Near Infrared Spectroscopy (fNIRS) to determine whether monitoring brain activity is an effective method for monitoring cognitive state. FNIRS is a portable optical neuroimaging technique that can be used to quantify hemodynamic activations. Moreover, it is relatively low-cost, non-confining, non-invasive, and safe for long-term monitoring (Boas et al., 2004; Gibson et al., 2005; Gratton et al., 2005; Steinbrink et al., 2005; Schroeter et al., 2006). Finally, temporal resolution is sub-second, and spatial resolution is on the order of 1 cm^2^ at best (Strangman et al., 2002a; Obrig and Villringer, 2003; Bunce et al., 2006). Measurements have been shown to be consistent with fMRI (Kleinschmidt et al., 1996; Strangman et al., 2002b; Steinbrink et al., 2005; Huppert et al., 2006; Schroeter et al., 2006; Emir et al., 2008) and electroencephalography (EEG) (Moosmann et al., 2003; Li et al., 2007). Critically, the ambulatory nature of fNIRS allows neuroimaging in the field. Thus, fNIRS may evolve into a synergistic complement to EEG and other physiological measures for monitoring cognitive state during operational tasks.

In the present study, we employed fNIRS to determine whether it is possible to distinguish between high and low levels of task engagement (i.e., performing a task vs. resting). Specifically, we monitored brain activity from the DLPFC in the “task-positive” network and from the MFG in the “task-negative” network while participants alternated between performing and not performing a cognitive task. We then employed multivariate pattern classification techniques in an effort to distinguish between periods of task performance and periods of rest.

To facilitate our ability to make this distinction, we asked participants to perform the multi-source interference task (MSIT; Stins et al., 2005; Bush and Shin, 2006). The MSIT is a selective attention task in which optimal performance requires participants to suppress multiple sources of interference (Stroop, Eriksen, and Simon). Thus, it reliably and robustly activates the “task-positive” network, even in individual task blocks from the same participant (Bush and Shin, 2006). Given these characteristics, we reasoned that the MSIT would provide a strong signal with which to monitor task engagement. Consistent with this reasoning, in the present study we were successful at distinguishing between relatively high and relatively low periods of task engagement.

We also conducted a companion fMRI study for verification and comparison purposes. First, given that DLPFC and MFG activity was recorded with fNIRS at the scalp surface, we wished to verify that our paradigm actually elicited hemodynamic activations in these regions. Second, we wished to compare fNIRS with fMRI with regard to the ability to distinguish between high and low levels of task engagement. Given that fMRI detects motor cortex activation reliably enough for routine use (Möller et al., 2005), and given that the motor cortex should be activated by the button presses required by our task, we expected that including such activation as an input to our classification algorithms would produce the highest levels of accuracy. Thus, we reasoned that classification accuracy with fMRI when including motor cortex activation would establish an “upper bound” for classification accuracy expectations. As expected, the fMRI study confirmed both of these predictions.

## Methods

### Behavioral methods

#### Participants

Seven participants (three females, four males) completed the fMRI study. Five participants (two female, three male) completed the fNIRS study (including two from the fMRI study). All participants practiced the behavioral task with performance feedback for 1 min at the beginning of the study. Next, they performed a set of four 7-min-long runs. Four runs each were completed for the fNIRS and fMRI experiments, which were performed on separate days. Human participant data were collected according to a protocol approved by both the University of Michigan IRB MED and the NASA IRB. Informed consent was obtained from all participants, who were healthy adults between the ages of 21 and 50 years. All participants were right-handed but one, who was ambidextrous.

#### Behavioral task

In each trial of the MSIT (duration, 2 s), participants viewed a horizontally-oriented array of four digits at the center of the screen (duration, 1 s). A target digit was printed in a larger font than each of three distracter digits (72 point vs. 60 point). The stimulus sizes were chosen to make the digits visible within the MRI scanner. Moreover, the large target digit was chosen to be about 20% larger than the small distracter digits to make the task sufficiently difficult. Indeed, Stins et al. (2005) observed a much smaller interference effect in the MSIT when the large target digit was 33% larger than the small distracter digits. Since the goal of the present MRI experiment was to contrast activity during task performance to activity during rest, we wanted to ensure that the task was difficult enough to elicit a good deal of task-related activity.

Participants were instructed to identify the target digit (1, 2, 3, or 4) by pressing a key with one of four fingers (1 = right thumb, 2 = right index finger, 3 = right middle finger, 4 = right ring finger) as quickly as possible without making mistakes. For congruent trials (50%), both the spatial position of the target and the identity of the three distracter digits were mapped to the correct response (1111, 2222, 3333, 4444). For incongruent trials (50%), both the spatial position of the target and the identity of the three distracter digits were mapped to a conflicting response (2111, 2122, 3343, 4443). Participants alternated between trials composed of 1s and 2s and trials composed of 3s and 4s across trials to prevent immediate stimulus and response repetitions (Mayr et al., 2003; Jiménez and Méndez, 2013).

Responses were recorded via the keyboard (the “n,” “u,” “9,” and “0” keys) of a laptop used in fNIRS experiments and via a MR-compatible response device in fMRI experiments. The task was implemented using a combination of MATLAB, 2012 (Natick, MA) and the Psychophysics Toolbox (Kleiner et al., 2007).

#### Functional neuroimaging experimental design

We employed a block design. In each of four runs, an initial 16 s rest block was followed by 12 alternations between the MSIT (16 s — 8 trials per block) and rest (16 s). Each run lasted 400 s.

### fNIRS methods

#### fNIRS data acquisition

**Figure 1:**
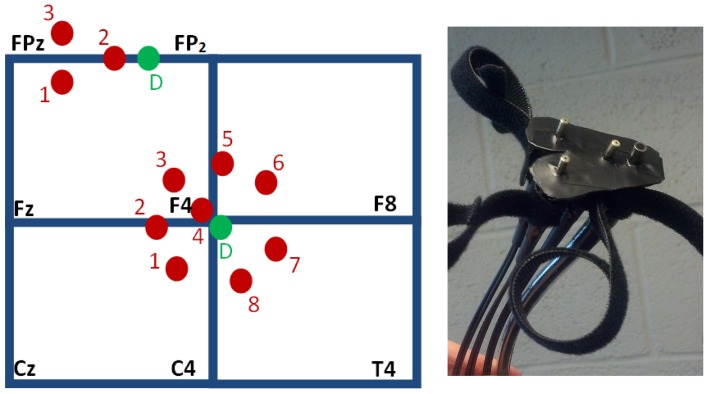
**Head probes used to interrogate the DLPFC and MFG. Left**: source channels (red) are arranged at a separation distance of 3 cm from two detectors (green, “D”), shown schematically with respect to the International 10–20 locations. The shallow sources, located at 1 cm from the detectors, provide channel 4 for the DLPFC array, which is near F4, and channel 2 for the MFG array, which is between FPz and FP_2_. The probes consistently producing the traces with the highest task model fit parameters were DLPFC probe channels 1–3 and MFG channel 1 (as numbered, **left**). **Right**: the skin-side of the array of head probes used for the MFG.

Hemoglobin concentration changes were measured using an Imagent NIRS instrument and fiber optic cables (ISS, Inc.). Eleven rigidly-connected source-detector pairs were used, and each source fiber delivered both 690 and 830 nm wavelength light. The data collection rate was 6.25 Hz. During each task run, 2500 time points were collected. Eight sources were located around one detector placed over the DLPFC region, and three sources were located around a second detector placed over the MFG. The array of head probes and fiber optic cables used to interrogate the MFG in this study is shown in Figure 1, right. The sources were held in place using both clear and blacked-out plastic at the locations shown with respect to the International 10–20 locations in Figure 1, left. The array of probes used to interrogate the MFG contained two sources placed 3 cm from the detector placed between FPz and FP_2_. The array of probes used to interrogate the DLPFC contained seven sources placed 3 cm from the detector placed near F4. The clear material (not shown) improved visual inspection for placement and hair control at the probe-skin interface. Blackout material was applied over and around the probes to block ambient light. Motor regions were not simultaneously interrogated due to instrumentation and fiber optic probe limitations.

The probes were located with the aid of an electroencephalography net (64-channel HydroCel Geodesic Sensor Net by EGI, Inc.) applied according to EGI Inc.'s instruction. The nets were used to identify the International 10–20 locations for each participant at each visit, not to record EEG data. A mark was made under the same net pedestals for all participants. The use of the nets made localization reliable, consistent and expeditious. The MFG mark was placed under a pedestal half way between FPz and FP_2_. This placed the sources for the MFG array (which were 2 cm from each other) at about the midline, with the detector then about 3 cm from the midline, to avoid the superior sagittal sinus as much as possible. The DLPFC mark was placed at the pedestal for EEG channel 59, which is immediately inferior to F4. The DLPFC array was placed by hand such that the detector and the source for channel 3 straddled its mark. The MFG array was placed similarly for channel 2. The probe arrays were secured with Velcro straps. The probes were not moved between the four runs unless the participant requested adjustment for the purposes of comfort. In those cases, care was taken to relieve pressure on the head without translating the probes.

Each probe array included a shallow source located 1 cm from the detector for superficial physiological and nuisance signal regression. The sources located 3 cm from the detectors provided deep traces of interest which sampled brain tissue, while the sources at 1 cm provided shallow traces which sampled only superficial tissue due to the proximity to the detector (Gagnon et al., 2011). The shallow channels were primarily sensitive to physiological changes in the skin, while also being sensitive to nuisance signal contributions such as motion of the rigidly-connected probe and ambient light exposure.

The Imagent instrument employs photomultiplier tubes for optical intensity detection. Gain settings for these were set on a per-participant basis after applying the probes and ensuring most of the hair was parted under the probe tips. Gain was increased and probes were re-adjusted to make better contact with the skin, iteratively, until as many channels as possible detected continuous wave intensity signal above a threshold of 500 analog-to-digital counts. None of the channels with low signal produced the best fit to the task model, and thus were not passed to the classification step (described below). The shallow channel sources at 1 cm may damage the detectors at gain settings appropriate for sources at 3 cm due to the lack of signal attenuation over their relatively shorter optical path length. To avoid this, delivered optical intensity was reduced for the shallow channel sources by layering optically absorbent pigment on partially-transmissive tape between the ends of the fiber probe tips and the skin.

#### fNIRS data processing

Both oxygenated and deoxygenated hemoglobin concentration ([Hb]) changes were calculated from the filtered raw continuous wave intensity measured traces using the Modified Beer Lambert Law (Delpy et al., 1998), then normalized (Huppert et al., 2009). Filtering was set to include 0.008–0.08 Hz to focus on sustained task activations while removing very slow drift and higher frequency physiological and motion contributions.

Standard linear regression was then used to remove physiological contributions from the measured signal to produce the functional task signal, and to select probes from each array. Probe channels, and specifically the oxygenated or deoxygenated [Hb] trace from that probe, were selected for use in classification based on how well the measured deep traces fit the expected functional activation task response, quantified by their beta fit parameter. The expected task response was modeled by convolving the boxcar task onset signal with the hemodynamic response function. The expected deoxygenated [Hb] time series was set to the negative of the oxygenated [Hb]. The shallow trace was smoothed using a 6-timepoint moving average, and task-like response in it was removed (by a separate regression step) prior to use as a nuisance regressor in the design matrix. The shallow trace removal was performed within-species. That is, the oxygenated [Hb] trace from the shallow channel was regressed from the measured oxygenated trace of each deep channel on that detector's array, and similarly for the deoxygenated traces. The functional task signal, which was effectively the measured signal minus the fitted nuisance signal, was then passed to the classification step.

Within- and across-network regional correlations, respectively, were defined as the time-series correlation coefficients relating activity between different regions of the DLPFC in the task-positive network and between the DLPFC and the MFG in the task-negative network. Each of these correlation values was averaged across all four runs in each participant, before it was averaged across participants. All statistical tests on correlation and accuracy values were one-tailed, with comparisons paired by participant.

#### fNIRS classification

**Figure 2:**
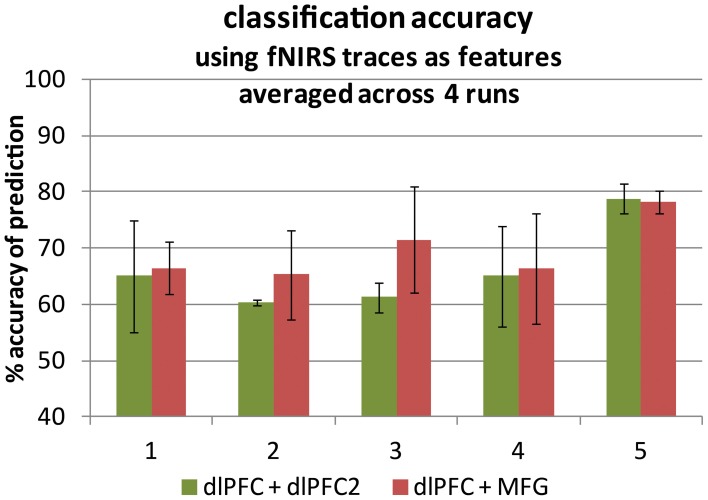
**Classification accuracy averaged across four runs for each of five participants.** Two fNIRS time traces were used as support vector machine input features in each case. **Left-hand bars**: within-network pairs (green). **Right-hand bars**: cross-region pairs (red). Accuracy is the number of time points for which the prediction matched the truth label out of all 2500 time points. An accuracy of 50% represents prediction by chance. Error bars represent ± one standard deviation.

**Figure 3:**
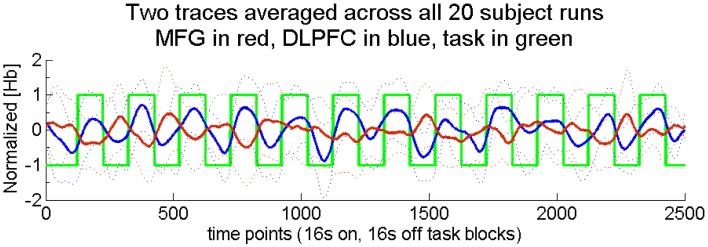
**Two across-region time traces averaged across all 20 runs.** These traces illustrate filtered, normalized, and corrected (see fNIRS methods) hemoglobin concentration changes. For the purposes of this group average, all deoxygenated traces were first inverted. Truth labels are indicated by the green trace with task at +1 and rest at −1. The across-network correlation for the group was −0.58. Task blocks were 16 s each, with equal rest time. Dotted lines show ± one standard deviation for each time point.

Classification was performed using two traces as input features to Support Vector Machines (SVM). Scripts were implemented in MATLAB, 2012 using LibSVM (Chang and Lin, 2011). We choose SVM for good performance with ease of implementation, processing speed in the interest of future real-time application, and the ability to use tuning parameters to optimize feature separation for each participant. First, the best two traces for each participant were selected from the array of seven deep DLPFC channels (a within-network pair, noted as DLPFC and DLPFC2 in Figure 2), based on which traces (oxygenated or deoxygenated) best fit the functional activation model as described above (which had the highest beta fit parameter considering all four runs). The traces which best fit the task model for each participant were assumed to make the best input features for producing the highest classification accuracy. The same best DLPFC trace was then paired with the best trace for each participant from the array of two deep MFG probes (an across-network pair, noted as DLPFC and MFG in Figures 2, 3) and a separate, second classification step was performed. Trace selections were not changed across runs.

A SVM model was trained to discriminate high from low levels of task engagement using three of each participant's four runs. Its prediction accuracy was tested on the participant's fourth run. Thus, training and prediction were always conducted within participants. All permutations were computed for each participant, such that the SVM model's prediction accuracy could be determined for each of the four runs. As described above, this was done separately for within- and across-network input pairs. The truth labels used for training and accuracy determination purposes were determined from the boxcar task onset signal, but shifted 4 s later to account for the delay of the hemodynamic response (the green trace in Figure 3).

A radial basis function kernel was employed for the SVM, which involves two tuning parameters. Kernel parameter gamma (g) determines the non-linearity of the mapping of our few features into a multidimensional space in which the prediction class labels are determined. A lower cost of error (c) allows more flexibility and generalizability of the SVM model by reducing the cost of misclassifications during model training (Chang and Lin, 2011). Non-linear mapping may improve performance depending on g. To determine the best possible prediction accuracy achievable with the methods of this study, c was tested at 0.1, 1, and 10, and g was tested at 0.001, 0.01, and 0.1 (a total of nine cases) in each classification step to optimize accuracy. These values were selected after running a test case varying c and g each across nine orders of magnitude. Accuracy did not change appreciably across four orders of magnitude, and the c, g values used were selected from this region. The highest classification accuracies produced using the same set of parameters across all four runs (the same c and g, given in Table 1) were selected for each participant and reported (see Figure 3). The best c, g parameters found upon optimization are not extreme in value and are not identical across participants. Accuracy was determined by comparing the work or rest state predicted for each time point to the known states (i.e., performing the task or resting).

**Table 1:** **The best *c*, *g* parameters found after optimization for each participant for fNIRS classification, and the time trace correlations (*r*) averaged across that participant's four runs**.

**Participant**	***c***	***g***	***r* (within)**	***r* (across)**
1	1	0.1	0.84	−0.21
2	10	0.001	0.23	−0.01
3	10	0.01	0.82	−0.16
4	10	0.1	0.95	−0.07
5	0.1	0.001	0.91	−0.15

### fMRI methods

#### fMRI data acquisition

fMRI data were acquired using a 3T GE Discovery MRI scanner with a T2^*^-weighted spiral BOLD sequence with pulse sequence parameters TR/TE/FA = 2 s/30 s/90°. The FOV was 22 cm in a 64 × 64 matrix for 40 slices with 3 mm thickness. The total time for each scan (400 s) was matched to the functional task at 200 volumes, after discarding 5 volumes at the beginning of each scan. Physiological signals were collected concurrently using a pulse oximeter and chest plethysmograph. For anatomical reference in the functional data analysis, a high-resolution T1-weighted anatomical image was collected using spoiled-gradient-recalled acquisition (SPGR) in steady-state imaging with pulse sequence parameters TR/TE/FA = 12.2 ms/5.2 ms/15°. The FOV was 26 cm in a 256 × 256 matrix for 136 slices at thickness 1.2 mm.

#### fMRI data processing

After slice-timing and motion correction, and physiological noise removal with RETROICOR (Glover et al., 2000), fMRI data were co-registered to the participant's anatomical scan, normalized to the MNI template (Collins et al., 1994), smoothed, modeled and estimated with SPM8 (Wellcome Department of Cognitive Neurology, London, UK). Smoothing was performed with a Gaussian kernel of 8 × 8 × 8 mm at full width half maximum. The resulting image files contained BOLD activation time traces for each voxel of the brain and were used in the classification step.

Both “rest minus task” and “task minus rest” contrasts were generated and used in second level random effects analyses performed across all runs for seven participants to inform fNIRS probe placement, and separately for each participant (across that individual participant's four runs) to guide voxel selection for fMRI classification.

Across-network, within-network and co-activating correlations were defined, respectively, as the correlation coefficients between across-network (i.e., the DLPFC and the MFG), within-network (i.e., DLPFC and DLPFC2), or co-activating (i.e., the DLPFC and motor cortex) pairs of functional task signals. The fMRI traces were additionally smoothed across 10 s to reduce the impact of noise in the signal, before the correlation coefficient was calculated. These were averaged across runs by participant, then averaged across participants.

#### fMRI classification

All fMRI traces used as SVM inputs were processed BOLD responses, averaged across clusters centered on local maxima within the regions of interest (DLPFC, primary motor cortex, and MFG). Eighteen voxels were symmetrically selected around participant-specific centers to be included in the average. Participant-specific locations were selected using the second-level statistical maps generated using that individual participant's four runs. The voxels used were not contiguous, and the region of interest spanned 1 cm per side in MNI space. In this way, the SVM input features for fMRI were restricted to traces averaged across local tissue. This is analogous, for fairness of comparison between the modalities, to the volume of tissue interrogated by one fNIRS probe, which is on the order of centimeters (Boas et al., 2004).

**Figure 4:**
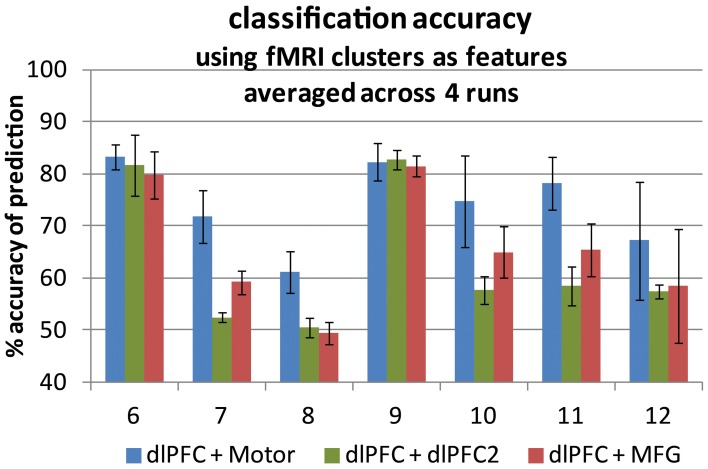
**Classification accuracy averaged across four runs for each of seven participants.** Two fMRI time traces were used as support vector machine input features in each case. Accuracy is the number of time points for which the prediction matched the truth label out of all 200 volumes. An accuracy of 50% represents prediction by chance. Error bars represent ± one standard deviation. **Left-hand bars**: co-activating pairs (blue). **Center bars**: within-network pairs (green). **Right-hand bars**: cross-region pairs (red).

Classification was performed with a fMRI trace selected from the contralateral motor area, which was paired with one from the DLPFC region (a co-activating pair, noted as DLPFC and Motor in Figure 4). The same DLPFC trace was paired with one from the MFG region (an across-network pair, noted as DLPFC and MFG in Figure 4), and classification was performed again. The same DLPFC trace was then paired with a second DLPFC trace from a region 2 cm superior and 2 cm medial to the first DLPFC trace in MNI space (a within-network pair, noted as DLPFC + DLPFC2 in Figure 4), and classification was performed a third time. To optimize accuracy, c was tested at 0.0001, 0.001, 0.01, 0.1, 1, 5, 10,100,1000, and g was tested at 0.00001, 0.0001, 0.001, 0.01,0.1,1, 5, 10, 100 (a total of 81 cases) in each classification step. Otherwise, all methods for classification were the same as those for the fNIRS traces described above. The SVM tuning parameters producing the best classification accuracies after optimization are given in Table 2. The accuracies are summarized in Figure 4.

**Table 2:** **The best *c*, *g* parameters found after optimization for each participant for fMRI classification, and the time trace correlations (*r*) averaged across that participant's four runs**.

**Participant**	***c***	***g***	***r* (co-acting)**	***r* (within)**	***r* (across)**
6	5	0.1	0.76	0.78	−0.23
7	1	1	0.06	0.06	0.10
8	0.1	0.01	0.57	0.48	0.34
9	1	1	0.72	0.85	−0.03
10	1	0.01	0.50	0.52	0.16
11	1	1	0.51	0.47	0.47
12	1000	0.00001	0.62	0.62	0.41

The motor region was selected for its robust and reliable response during task periods. We treat classification based on time traces from the motor region as a gold standard. That is, we do not expect fNIRS classification accuracies to exceed those attainable using motor cortex activations measured with fMRI.

## Results

### Behavioral task results

As described above, five participants completed the fNIRS study and seven participants completed the fMRI study. One participant performed the task incorrectly for incongruent trials. Thus, this participant's data were excluded from the behavioral analyses. However, since this participant was engaged in the task and responding to stimuli, these trials were not excluded from the fNIRS and fMRI analyses. Including these data was appropriate because the fNIRS and fMRI analyses were aimed at distinguishing between performing a task and resting, rather than distinguishing between incongruent and congruent trials. Mean accuracy was 98% (*SD* = 2.2%, *N* = 4) for the four fNIRS participants who performed the task correctly, and 97% (*SD* = 4.7%, *N* = 6) for the six fMRI participants who performed the task correctly. As expected, mean accuracy was relatively high.

Also as expected, performance was worse in incongruent than in congruent trials (the analysis of the data included only the participants who performed correctly in most incongruent trials). In a random effects analysis, mean reaction time was significantly higher in the incongruent condition (fNIRS: *M* = 0.639 s, *SD* = 0.035, *N* = 4; fMRI: *M* = 0.789 s, *SD* = 0.114 s, *N* = 6) than in the congruent condition (fNIRS: *M* = 0.552 s, *SD* = 0.033 s, *N* = 4; fMRI: *M* = 0.714 s, *SD* = 0.096 s, *N* = 6), [fNIRS: *t*_(3)_ = 26, *p* < 0.0005; fMRI: *t*_(5)_ = 9.1, *p* < 0.0005]. Likewise, mean error rate was significantly higher in the incongruent condition (fNIRS: *M* = 2.0%, *SD* = 1.8%, *N* = 4; fMRI: *M* = 4.9%, *SD* = 6.4%, *N* = 6) than in the congruent condition (fNIRS: *M* = 0.38%, *SD* = 0.81%, *N* = 4; fMRI: *M* = 2.1%, *SD* = 3.5%, *N* = 6), [fNIRS: *t*_(3)_ = 2.9, *p* < 0.05; fMRI: *t*_(5)_ = 2.2, *p* < 0.05). Errors of omission were rare and thus not analyzed.

### fNIRS results

The four-run average classification accuracy for each of five participants is presented in Figure 2. As expected, we were able to distinguish between task engagement and rest. In particular, both averages differed significantly from chance at 50% [across: *t*_(4)_ = 9.65, *p* < 0.0005; within: *t*_(4)_ = 4.95, *p* < 0.005]. Also in line with predictions, there was a non-significant trend toward greater classification accuracy for across-network pairs (*M* = 69.1%, *SD* = 4.4%) than for within-network pairs (*M* = 66.0%, *SD* = 7.2%), [*t*_(4)_ = 1.48, *p* < 0.25]. The probe channels resulting in oxygenated or deoxygenated [Hb] traces with the highest task model fit parameters were 1, 2 and 3 (as numbered in Figure 1, left; data not shown). The [Hb] species of the best-fitting traces were nearly evenly split: eight were oxygenated and seven were deoxygenated [Hb] traces. Thus, we found no universally best Hb species for fitting the task model. This result is consistent with prior suggestions that a probe's sensitivity to one species or the other depends on whether it mostly samples the arterial or venous compartment (Strangman et al., 2003), which is likely to change for every probe application.

Prior work indicates that activity in key regions of the “task-positive” network is positively correlated while activity in key regions of the “task-positive” and “task-negative” networks is negatively correlated (Fox et al., 2005; Kelly et al., 2008). Consistent with such findings, the group-averaged correlations for within-network pairs were significantly greater than zero [*r* = 0.75, *SD* = 0.29; *t*_(4)_ = 5.72, *p* < 0.005], while those for across-network pairs were significantly less than zero [*r* = −0.12, *SD* = 0.08; *t*_(4)_ = 3.41, *p* < 0.025]. Further, the correlation averages for within-network pairs were significantly higher than those of the across-network pairs [*t*_(4)_ = 5.56, *p* < 0.005; see Table 1 for a participant-specific list of correlation values]. These findings suggest that our fNIRS probes accurately measured activity in the “task-positive” and “task-negative” networks.

Finally, since across-network correlation was determined on a per-participant basis, we also wished to verify that negatively correlated across-network activity was observed at the group level. To this end, we averaged the across-network functional task signals across all 20 runs. For the purposes of this group average, all deoxygenated traces were first inverted, consistent with a reduction of deoxygenated [Hb] during activation. The group-averaged time traces are presented in Figure 3, which shows filtered, normalized and corrected [Hb] changes [see fNIRS methods; truth labels (green trace) show task at +1 and rest at −1]. As expected, the across-network correlation determined in this fixed effects analysis was significantly less than zero [*r*_(18)_ = −0.58, *p* < 0.01]. This finding illustrates that, even at the group level, DLPFC activity (blue trace) increased during task performance while MFG activity (red trace) decreased.

### fMRI results

The four-run average classification accuracy for each of the seven participants is presented in Figure 4. Replicating the fNIRS results, group averaged classification accuracy was significantly greater than chance at 50% for all three types of region pairs [across: *t*_(6)_ = 3.56, *p* < 0.01; within: *t*_(6)_ = 2.56, *p* < 0.025; co-activating: *t*_(6)_ = 7.96, *p* < 0.0005], indicating we were able to distinguish task engagement from rest. Also as expected, group averaged classification accuracy was significantly higher for the co-activating (DLPFC and motor) pairs (*M* = 74.1%, *SD* = 8.0%) than for the across-network pairs (*M* = 65.6%, *SD* = 11.6%), [*t*_(6)_ = 4.83, *p* < 0.005], consistent with the high reliability of motor cortex activation detection in fMRI studies (Möller et al., 2005) and with the motor cortex activation associated with button press responses in the present task. Within-network pair accuracy (*M* = 63.0%, *SD* = 13.5%) tended to be lower than across-network pair accuracy, but not significantly [*t*_(6)_ = 1.57, *p* < 0.1].

Also consistent with the fNIRS results, the group-averaged correlations for co-activating (*r* = 0.53, *SD* = 0.23) and within-network pairs (*r* = 0.54, *SD* = 0.26) were significantly greater than zero [within: *t*_(6)_ = 6.07, *p* < 0.0005; co-activating: *t*_(6)_ = 5.49, *p* < 0.005]. In contrast, those for the across-network pairs (*r* = 0.17, *SD* = 0.25) did not differ from zero [*t*_(6)_ = 1.81, *p* < 0.1]. As predicted, however, they were significantly lower than those for the within-network pairs [*t*_(6)_ =2.29, *p* < 0.05; see Table 2 for a participant-specific list of correlation values].

**Figure 5:**
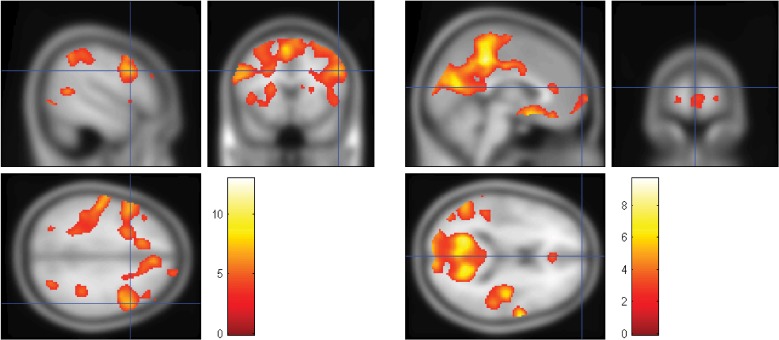
**The locations of statistically significant activations for the MSIT, after second-level analysis across seven participants.** The expected “task-positive” and “task-negative” hemodynamic activations occurred in the same regions that were interrogated by the fNIRS probes. **Left**: the *t*-statistic is mapped, with the height threshold set at an uncorrected significance level of *p* < 0.001 and the extent threshold set at 10 voxels. DLPFC is shown at [52, 14, 32] as marked by the crosshair in MNI space for the work minus rest contrast. **Right**: the *t*-statistic is mapped, with the height threshold set at an uncorrected significance level of *p* < 0.01 and the extent threshold set at 10 voxels. MFG is shown at [22, 66, 0] in MNI space for the rest minus work contrast. (Crosshair: *x* = 0, *y* = 66, *z* = 14).

The locations of statistically significant activations for the MSIT, after second-level analysis across four runs each from seven independent participants, are shown for the DLPFC [(52, 14, 32) in MNI space; Figure 5, left] and for the MFG [(22, 66, 0) in MNI space; Figure 5, right]. The *t*-statistic is mapped, with the threshold set at an uncorrected significance level of *p* < 0.001 for the work minus rest contrast shown on the left, and at *p* < 0.01 for the rest minus work contrast shown on the right. The MFG activation was not present at the higher threshold but appeared at the lower threshold. Of importance, the expected “task-positive” and “task-negative” hemodynamic activations occurred in the same regions that were interrogated by the fNIRS probes. Notably, even with only seven participants, the DLPFC survived a family wise error correction at *p* < 0.05; the MFG, however, did not survive this correction.

Finally, we note that although other regions of the “task-negative” network were identified in the fMRI analysis, they may be less useful for monitoring task engagement with fNIRS. First, lateral parietal regions were not consistently activated bilaterally across participants. Thus, monitoring both sides with fNIRS would present greater difficulty due to the increased number of optical probes. Second, although precuneus and PCC regions of the task-negative network (Raichle et al., 2001; Greicius et al., 2003) were reliably activated (Figure 5, right), they are too deep to be accessible via fNIRS probes, which can interrogate only the outer layers of the cortex (Boas et al., 2004).

## Discussion

In the present study, we investigated whether functional neuroimaging methods (i.e., fNIRS and fMRI) can be employed to distinguish periods of task engagement from periods of rest. As described below, our findings support this view. They also provide valuable information about which brain activations may prove most useful for monitoring task engagement in the field.

Our first set of findings came from fNIRS experiment. Here, we found that multivariate pattern classification techniques could distinguish between periods of task performance and periods of rest based on brain activity recorded from (a) different regions of the DLPFC in the task-positive network (a within-network pair) or (b) the DLPFC in the task-positive network and the MFG in the task-negative network (an across-network pair). Further, there was a trend toward higher classification accuracy for across-network pairs than for within-network pairs. Indeed, accuracy with across-network pairs approached 70%, even with the basic processing methods described here (with adaptive physiological filtering and additional probes, accuracy may further improve). This result fits with prior data suggesting that variability in task engagement is associated with variability in activity and/or functional connectivity involving both the “task-positive” and the “task-negative” networks (e.g., Weissman et al., 2006; Prado and Weissman, 2011). Most important, our fNIRS findings indicate that online recordings of brain activity via fNIRS may provide a valuable tool for detecting varying levels of task engagement in the real world.

Our second set of findings came from an fMRI study. Of importance, these findings both verified and extended the results of the fNIRS study discussed earlier. First, we observed activations and deactivations, respectively, in the DLPFC and the MFG, which verified that our functional neuroimaging paradigm engaged the task-positive and task-negative networks. Second, further analyses revealed that these activations occurred in the same DLPFC and MFG regions that were activated in the fNIRS experiment, wherein activity was measured with probes on the scalp. Third, the trend toward higher classification accuracy for across-network pairs than for within-network pairs observed with fNIRS was also observed with fMRI. Fourth, the fMRI findings indicated a possible upper bound on classification accuracy for distinguishing between task performance and rest: as expected, the highest classification accuracy was observed when the DLPFC trace and co-activating (for this task) motor cortex trace served as inputs to the SVM classifier. Together, these fMRI findings verified that our fNIRS recordings reflected activity in the key regions under investigation (DLPFC and MFG). They also replicated the fNIRS results and extended them by suggesting an upper bound for classification accuracy based on relatively focal hemodynamic activity.

### Novel contribution of the present work

The present findings make an important contribution to the field. Specifically, they show, for the first time, that it is possible to detect negative correlations between activity in key regions of the “task-positive” and “task-negative” networks with fNIRS. Further, they show that the detection of activity in the “task-negative” network is useful for distinguishing between high and low levels of task engagement. This capability might prove useful in future applications of fNIRS that are aimed at discriminating between optimal behavioral performance (where a negative correlation is expected) and internally-guided thought (where co-activation and, hence, a positive correlation is expected) (Christoff et al., 2009; Smallwood et al., 2012). Thus, it could function to improve the predictive power of a fNIRS-based cognitive state monitoring system. Finally, although our findings make a novel contribution to the field, it is important to note that they build on previous work showing that frontal oxygenation is sensitive to workload (Izzetoglu et al., 2004) and that fNIRS can reliably detect both resting state physiology and functionally-connected networks (White and Culver, 2008; Mehnert et al., 2009; Mesquita et al., 2010).

### Limitations

While across-network pairs were associated with stronger negative correlations and higher classification accuracy, relative to within-network measures (data not shown), negative correlations between DLPFC and MFG activity were not observed in every participant. This lack of consistency may stem from a variety of sources, including non-optimal fNIRS probe localization, variation in participant compliance or strategy, interference from physiological or motion artifact, variable fMRI voxel selection, and co-activation of key regions in the “task-positive” and “task-negative” networks during mind wandering or internally-guided thought (Christoff et al., 2009; Smallwood et al., 2012). Future studies should be conducted to distinguish among these possibilities and to determine which methodologies provide more consistent measures of negatively correlated activity in the task-positive and task-negative networks.

Also regarding the consistency of our measures, classification accuracy varied considerably across runs (see the error bars in Figures 2, 4). Future studies might therefore be conducted to investigate the source(s) of this variability as well as the impact of other sources of variability (e.g., across-visit, across-participant and across-task) on classification accuracy. Such studies might also investigate the impact of using the known task model to clean the measured traces when producing functional task signals for use in classification (see fNIRS data processing), which may have biased the classifier toward higher accuracy in the present study.

Another study limitation stems from the fact that some task-evoked systemic signals are measureable on the scalp surface, and that at least one such signal—skin blood volume—depends on cognitive state (Kirilina et al., 2012). Since fNIRS is sensitive to hemodynamics in superficial tissue at all source-detector separation distances, it is possible that systemic signals in the superficial tissue may have driven the negative across-network correlations that we observed. To investigate this possibility, we quantified correlations for the traces taken from the across-network shallow source-detector pairs (see fNIRS data acquisition). Of importance, no association was observed between the correlation values for the superficial traces and the correlation values for the deep traces, whether corrected or not (data not shown). Thus, the negative correlations that we measured between the across-network deep traces likely reflected [Hb] related changes in brain tissue rather than superficial physiological signals. However, if skin blood changes provide additional information about the task engagement, then it could be useful in future studies to include such changes directly as classifier inputs.

Finally, we note that motor activation is not always a reliable component of task engagement as some tasks require sustained attention over long periods in the absence of overt responses (e.g., instrument cross-checking and visual display searches). Thus, future studies may wish to focus on our fNIRS finding that classification accuracy was slightly higher for across-network pairs (i.e., pairs in which one region came from the task-positive network while the other came from the task-negative network) than for within-network pairs (i.e., pairs in which both regions came from the task-positive network). As we mentioned earlier, this finding fits with the view that task engagement is determined by interactions between these networks (Fox et al., 2005; Weissman et al., 2006; Kelly et al., 2008; Prado and Weissman, 2011).

### Future work

Future work could address whether adaptive filtering of fNIRS traces over smaller time windows improves the activation-based classification measures reported here. This may include investigating adaptive physiological noise removal driven by the correlation between the deep and shallow traces (Harrivel et al., 2012) or motion artifact reduction based on the frequency domain phase signal (Harrivel and Hearn, 2012). An increase in probe density could also be used to improve localization within the regions of interest. Finally, measures of network correlation could be quantified over shorter time scales to determine whether transient internally-guided thought can be distinguished from periods of “zoning out.” Ongoing simultaneous fNIRS/fMRI studies are further examining these and other possible methods for improving our ability to discriminate between varying levels of task engagement.

## Conclusion

In the present study, we used a combination of fNIRS and fMRI results to show that online recordings of brain activity from the task-positive and task-negative networks can be used to detect moment-to-moment changes in task engagement. We hope that future studies combining fNIRS recordings with multivariate classification methodologies will build upon the present work to produce robust systems for detecting changes in task engagement in real-world settings.

## Authors contributions

Angela R. Harrivel is the PI on the human subject study. She recruited and consented participants, designed fNIRS head probes, wrote all scripts for the fNIRS data processing, collected and processed all data, performed analyses and wrote the manuscript. Daniel H. Weissman wrote the code for the MSIT presentation, provided guidance regarding psychological aspects of the study and fMRI analyses, and edited the manuscript. Douglas C. Noll provided funding support, guidance regarding all fMRI aspects of the project and all data processing methods, and reviewed the manuscript. Scott J. Peltier is Co-I on the study. He contributed to analyses, edited the manuscript, and provided guidance regarding data collection and processing techniques, pattern classification methods, and resting state analyses.

### Conflict of interest statement

The authors declare that the research was conducted in the absence of any commercial or financial relationships that could be construed as a potential conflict of interest.
